# Application of lymphoplasmapheresis in the treatment of severe myasthenia gravis

**DOI:** 10.3389/fneur.2022.1018509

**Published:** 2022-10-11

**Authors:** Weiwei Duan, Hao Zhou, Xiaohua Dong, Bijuan Li, Yi Li, Haobing Cai, Qian Zhou, Song Ouyang, Weifan Yin, Huan Yang

**Affiliations:** ^1^Department of Neurology, Xiangya Hospital, Central South University, Changsha, China; ^2^Department of Blood Transfusion, Xiangya Hospital, Central South University, Changsha, China; ^3^Department of Neurology, The Affiliated Changsha Hospital of Xiangya School of Medicine, Central South University, Changsha, China; ^4^Department of Neurology, The Second Xiangya Hospital, Central South University, Changsha, China

**Keywords:** myasthenia gravis, lymphoplasmapheresis, immunotherapy, efficacy, safety

## Abstract

**Background:**

Lymphoplasmapheresis (LPE) is a treatment that combines traditional plasma exchange and lymphocyte removal technique. It has been applied to treat a variety of autoimmune diseases, but its application value in the treatment of severe myasthenia gravis (MG) is not yet clear. Therefore, the aim of this study was to investigate the efficacy and safety of LPE in severe MG.

**Methods:**

Clinical data of 123 severe patients with MG (Myasthenia Gravis Foundation of America Clinical Classification, Class IV) who received LPE treatment were included in a retrospective analysis. Efficacy was evaluated by the change of Quantitative Myasthenia Gravis score (QMGS) before and after treatment. Univariate and multivariate logistic regression analysis was used to explore clinical factors affecting efficacy.

**Results:**

A total of 220 replacements were performed in 123 patients, with an average of 1.79 replacements per patient. The overall safety of LPE was good, and no serious adverse reactions occurred. After treatment, the mean QMGS of patients decreased significantly, from 23.40 ± 4.25 points before treatment to 17.93 ± 5.61 points after treatment, a decrease of 5.47 ± 4.16 points. 75.6% of patients experienced remission of clinical symptoms. During a 2-month follow-up of 64 patients, a progressive improvement in QMGS was found. Each muscle group involved in MG responded well to LPE treatment. In addition, LPE significantly reduced the levels of AChR-Ab and inflammatory cytokines in patients. Age ≥ 50 years and co-infection were unfavorable factors affecting the efficacy.

**Conclusions:**

In this study cohort, LPE is safe for the treatment of severe MG and achieves good treatment outcome with fewer replacements. In patients with MG, the avoidance and timely control of infection are necessary. Our study provides a potential new treatment option for severe MG.

## Introduction

Myasthenia gravis (MG) is an acquired autoimmune disease involving the neuromuscular junction with a prevalence of 15–179 per million people (1). Acetylcholine receptor (AChR), muscle specific kinase (MuSK), and low-density lipoprotein receptor-related protein 4 (LRP4) are pathological targets in MG (2). Certain patients with MG have additional autoantibodies (Abs) against muscle-associated proteins such as titin and ryanodine receptor (RyR), although their pathophysiological relevance is obscure (3). MG is characterized by fluctuating skeletal muscle fatigue, usually first involving the extraocular muscles (ocular myasthenia gravis, OMG), manifesting as ptosis and diplopia. Most patients progress over several years, gradually involving other skeletal muscles (generalized myasthenia gravis, gMG), resulting in limb weakness, dysphagia, dysarthria, and even fatal respiratory muscle paralysis (myasthenic crisis) (4). In addition, thymus abnormalities (thymic hyperplasia or thymoma) have been associated with the development of some MG entities (5).

Currently, the treatment strategies for MG mainly include symptomatic treatment and immunotherapy for pathological immune responses. Corticosteroids, azathioprine (AZA), tacrolimus, and mycophenolate mofetil (MMF) are commonly used immunosuppressants in long-term immunomodulatory therapy (6). Thymectomy is appropriate for patients with thymoma and non-thymomatous patients with AChR-Ab positive, gMG, and aged 18–50 years (7, 8). In addition, thymectomy should be considered in AChR-Ab-positive patients with gMG who have a poor response to adequate immunosuppressive therapy or have intolerable side effects from that therapy (8). Patients with severe MG are not only severely impaired in their daily living ability, but also may experience myasthenic crisis, which is life-threatening. Hence, it is essential to improve their symptoms rapidly. Intravenous immunoglobulin (IVIG) and plasma exchange (PE) are currently the first-line treatment option for patients with severe MG to obtain a rapid clinical response (9, 10). PE may be a better option with faster onset and better efficacy (7).

Lymphoplasmapheresis (LPE) is developed on the basis of traditional PE combined with lymphocyte removal technology. In contrast to PE, it not only eliminates circulating soluble pathological substances in plasma, such as autoantibodies, complement, cytokines, and adhesion molecules, but also removes immunocompetent cells, such as sensitized T and B lymphocytes (11). At present, LPE has been applied in the treatment of various autoimmune diseases. In refractory rheumatoid arthritis, LPE has shown better therapeutic effect than traditional PE (12, 13). For acute renal allograft rejection, LPE further potentiates immunosuppression effect compared with PE (14). In addition, LPE has been successfully used in the treatment of refractory rare autoimmune skin diseases, such as acute generalized pustular psoriasis of von Zumbusch and febrile ulceronecrotic Mucha-Habermann disease (15, 16). In autoimmune diseases of the nervous system, LPE has demonstrated favorable efficacy against Guillain-Barre syndrome and steroid-refractory neuromyelitis optica spectrum disorders (17, 18). However, little is known about whether LPE is applicable to severe MG. Therefore, this study conducted a retrospective analysis on the clinical data of patients with severe MG who received LPE treatment in our center to evaluate its efficacy and safety.

## Methods

### Data sources

We collected the clinical data of 146 patients with severe MG who received LPE in the Department of Neurology, Xiangya Hospital from November 2016 to January 2022. The diagnosis of MG was made based on the symptoms of fluctuating and fatigable muscle weakness combined with a positive test for specific autoantibodies. In antibody negative cases, repetitive nerve stimulation and neostigmine tests were used to ensure diagnosis (19). In this study, severe MG was defined as MG patients with Myasthenia Gravis Foundation of America (MGFA) Class IV (20). A detailed description of MGFA clinical classification can be found in Supplementary Table 1. Twenty-three patients were excluded from this study due to lack of post-treatment quantitative myasthenia gravis score (QMGS) (21). Finally, 123 patients were included in the study. Clinical data collected included baseline demographic and clinical characteristics before treatment, treatment-related side effects, and QMGS after treatment. In addition, we collected data on immune-related indicators before and after treatment (missing in some patients), including AChR-Ab (*n* = 76), IgG (*n* = 87), Interleukin-1β (IL-1β, *n* = 82), IL-6 (*n* = 82), and tumor necrosis factor-α (TNF-α, *n* = 82) levels, and lymphocyte counts (*n* = 101). The collection time points of immune indexes were within 3 days before treatment and within 3 days after the completion of the entire LPE treatment. This study protocol was approved by The Ethics Committee of Xiangya Hospital.

### LPE procedure

LPE was implemented according to previously reported methods (11, 16, 17). Briefly, LPE was performed through peripheral veins using a blood cell separator (COBE Spectra, USA). The leukocyte channel was installed, and the lymphocytes were collected by photoelectric colorimetric technology and density gradient centrifugation in a manual program. The reinfusion channel was clamped to allow the autologous plasma to enter the collection bag and be removed together with the lymphocytes, while the fresh frozen plasma was injected from the clamp at the top left of the heparin cap and reinfused into the patient's body after confluence with the patient's red blood cells. In each replacement, ~70–80% of the total plasma volume (TPV) was exchanged and replaced with an equal volume of fresh frozen plasma, and 2–3 × 10^∧^9 lymphocytes were removed. The total number and concentration of removed cells were monitored and the parameters were adjusted dynamically. TPV was calculated using the following formula: TPV = 0.065 × (1-hematocrit) × weight (kg). The LPE course consisted of 1–3 procedures, once every 3 days.

### Synchronous therapy regimen

Long-term immunosuppressive maintenance therapy was given concurrently during LPE treatment. For patients not currently receiving immunotherapy, low-dose oral corticosteroids (prednisone 20 mg per day, plus 5 mg every 3 days to maintain at 1 mg/kg per day) and/or immunosuppressants (AZA, tacrolimus, and MMF) were given. The initial dose of tacrolimus was 1 mg each time, twice a day; the initial dose of AZA was 50 mg per day; the initial dose of MMF was 0.5 g each time, twice a day. For patients currently receiving immunotherapy, the current regimen was maintained or adjusted as appropriate based on serum concentration, drug tolerance and responsiveness. The use of acetylcholinesterase inhibitors (AChEIs) such as pyridostigmine bromide was permitted. None of the patients received high-dose corticosteroid pulse therapy in the LPE treatment phase. In addition, the patients with co-infection were treated with sensitive antibiotics at the same time, and no patients had worsening of infection, which were effectively controlled. The use of oral immunomodulatory drugs during LPE treatment is summarized in Table 1.

**Table 1:** Baseline clinical characteristics of patients with severe myasthenia gravis.

**Baseline characteristics**	
Male/female (*n*, %)	47 (38.2%)/76 (61.8%)
Age (years, mean ± SD)	45.24 ± 16.12
Disease duration (months, mean ± SD)	46.64 ± 71.28
**Thymus condition (** ***n*** **, %)**	
Thymoma	25 (20.3%)
Thymic hyperplasia	4 (3.3%)
Normal	94 (76.4%)
**History of thymectomy (** ***n*** **, %)**	
Yes	28 (22.8%)
No	95 (77.2%)
**Autoimmune disorders (** ***n*** **, %)**	
Autoimmune thyroid disorders	23 (18.7%)
Rheumatoid arthritis	1 (0.8%)
No	99 (80.5%)
**Co-infection (** ***n*** **, %)**	
Respiratory infection	28 (22.8%)
Urinary tract infection	2 (1.6%)
No	93 (75.6%)
**Immunotherapy before treatment**	
Yes	66 (53.7%)
No	57 (46.3%)
**History of myasthenic crisis**	
Yes	14 (11.4%)
No	109 (88.6%)
**QMG score (mean** **±SD)**	23.40 ± 4.25
**MGFA classification (** ***n*** **, %)**	
IVa	22 (17.9%)
IVb	101 (82.1%)
**AChR-Ab (** ***n*** **, %)**	
Positive	110 (89.4%)
Negative	13 (10.6%)
**MuSK-Ab (** ***n*** **, %)**	
Positive	4 (3.3%)
Negative	119 (96.7%)
**LRP4-Ab (** ***n*** **, %)**	
Positive	0 (0%)
Negative	123 (100%)
**Titin-Ab (** ***n*** **, %)**	
Positive	26 (21.1%)
Negative	97 (78.9%)
**RyR-Ab (** ***n*** **, %)**	
Positive	15 (12.2%)
Negative	108 (87.8%)
**Oral immunomodulatory drugs (** ***n*** **, %)**	
Prednisone	9 (7.3%)
Prednisone and tacrolimus	88 (71.5%)
Prednisone and mycophenolate mofetil	10 (8.1%)
Prednisone and azathioprine	5 (4.1%)
Tacrolimus	8 (6.5%)
Mycophenolate mofetil	3 (2.4%)
Length of hospital stay (days, mean ± SD)	11.26 ± 4.19
Hospitalization costs (CNY, mean)	28,779.15

### Efficacy evaluation

QMGS was applied for efficacy evaluation. It is a clinical scoring system for assessing disease severity in patients with MG, measuring 13 objective parameters on a scale of 0 to 3, with 3 being the most severe, with a total score ranging from 0 to 39. A reduction of ≥3 points in QMGS after treatment was used as the threshold for treatment response (21). The patients were routinely assessed for QMGS within 3 days before the start of LPE treatment and within 3 days after each LPE (the second or third or fourth day after treatment). For patients who received multiple LPE, the pre-treatment and last scores were selected for efficacy analysis. In order to exclude the effect of AChEIs on QMGS, pyridostigmine bromide was discontinued for 6–8 h before each score.

### Safety evaluation

All reports of adverse events related to LPE were collected to assess safety. Adverse events in this study were defined as any adverse medical event that occurred after a patient received LPE, which could be symptoms, signs, or laboratory abnormalities. Serious adverse events refer to adverse medical events such as death, life-threatening conditions, permanent or severe disability and functional impairment, and prolonged hospitalization in patients following LPE treatment.

### Statistical analysis

SPSS software (version 26.0) was used for statistical analysis in this study. Continuous data were expressed as mean ± standard deviation (SD). Categorical variables were expressed as counts and percentages. Paired *t*-test were used to compare QMGS and immune indexes before and after treatment. Repeated measures analysis of variance (RMANOVA) was used to assess changes in QMGS during follow-up. Univariate and multivariate logistic regression were applied to identify clinical factors associated with efficacy. In this study, *p* < 0.05 was considered statistically significant.

## Results

### Patient characteristics

**Figure 1:**
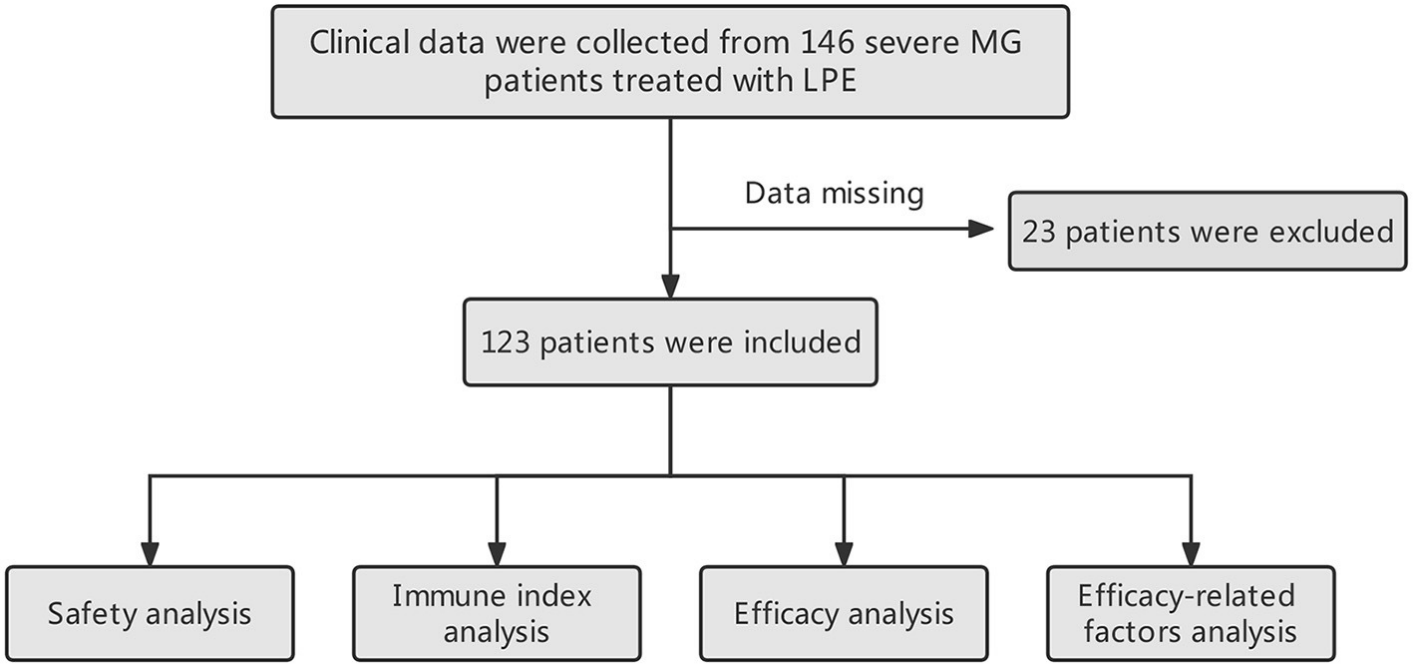
The flowchart designed for this study.

The workflow designed for the study is shown in Figure 1. A total of 123 patients with severe MG who received LPE were enrolled in this study, including 22 cases with MGFA IVa and 101 cases with MGFA IVb. There were 47 males, accounting for 38.2%; 76 females, accounting for 61.8%. The average age was 45.24 ± 16.12 years (range 18–80 years). The average disease duration was 46.64 ± 71.28 months. Thymus abnormalities occurred in 23.6% of patients, including 25 (20.3%) cases with thymoma and 4 (3.3%) cases with thymic hyperplasia. Twenty-eight (22.8%) patients had a history of thymectomy. 19.5% (*n* = 24) of patients were complicated with other autoimmune diseases, with autoimmune thyroid disorders being the most common. The incidence of co-infection was 24.4% (*n* = 30), mainly respiratory infection. 53.7% (*n* = 66) of patients were in process of receiving regular immunotherapy before treatment. Fourteen (11.4%) patients had a history of myasthenic crisis. The mean QMGS before treatment was 23.40 ± 4.25 points. In terms of MG-related Abs, AChR-Ab was positive in 110 cases (89.4%) and negative in 13 cases (10.6%); MuSK-Ab was positive in 4 cases (3.3%) and negative in 119 cases (96.7%); 26 (21.1%) patients were positive for titin-Ab and 15 (12.2%) patients were positive for RyR-Ab; No LRP4-Ab positive patients were found. In addition, the average length of hospital stay was 11.26 ± 4.19 days, and the average hospitalization costs was 28,779.15 CNY. Table 1 summarized the patients' baseline characteristics.

### Efficacy of LPE

**Figure 2:**
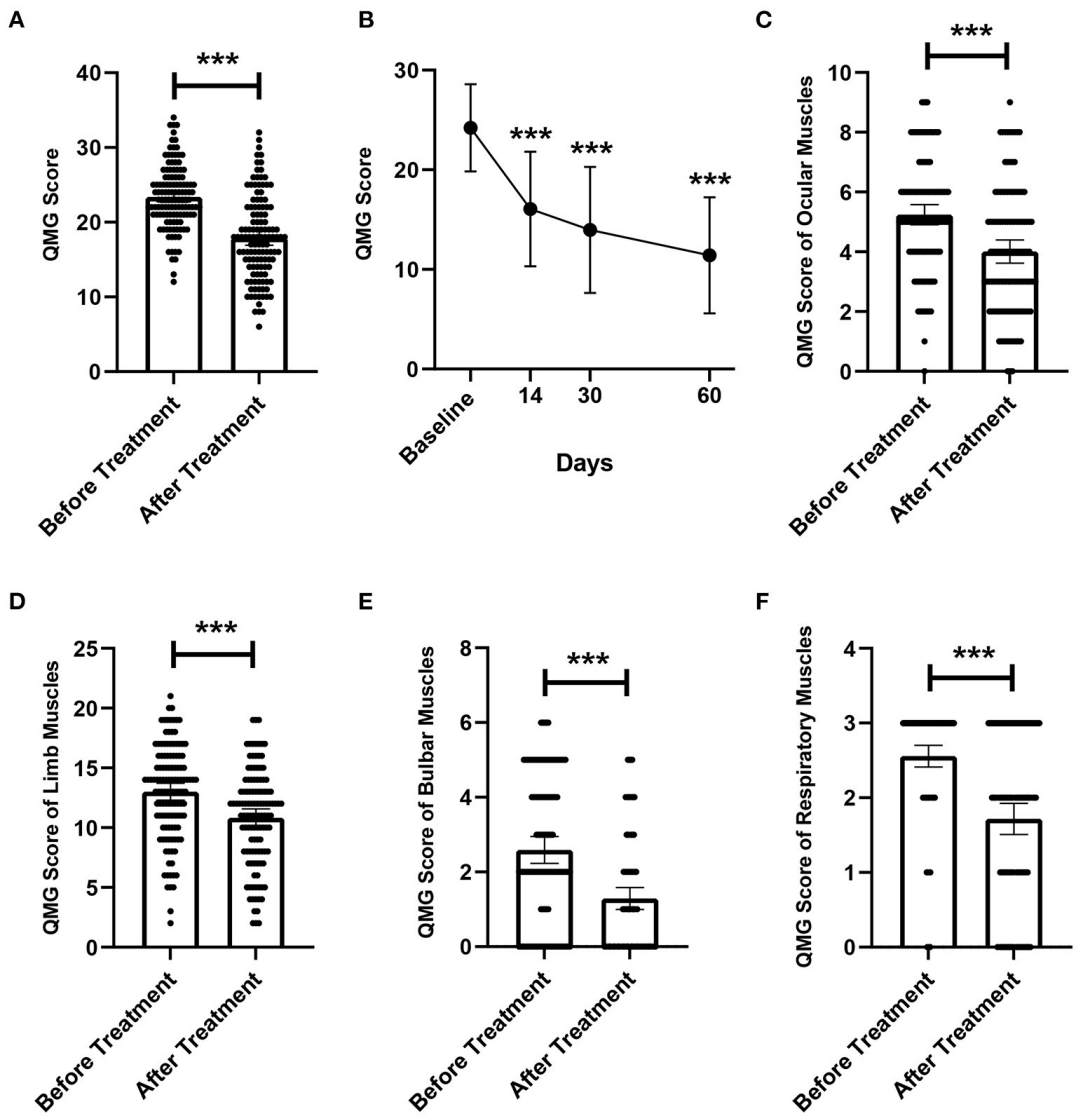
Changes in QMGS before and after treatment. **(A)** Changes in overall QMGS before and after treatment. **(B)** Changes in QMGS of patients before treatment, 14, 30, and 60 days after treatment completion. **(C)** Changes in QMGS of ocular muscles before and after treatment. **(D)** Changes in QMGS of limb muscles before and after treatment. **(E)** Changes in QMGS of bulbar muscles before and after treatment. **(F)** Changes in QMGS of respiratory muscles before and after treatment. ***p < 0.001.

A total of 220 replacements were performed in 123 patients, of which 28 patients received one replacement, 93 patients received two replacements, and 2 patients received three replacements, with an average of 1.79 times. We evaluated the efficacy of LPE by comparing the changes in QMGS before treatment and after the completion of the entire LPE treatment. The mean QMGS of patients before treatment was 23.40 ± 4.25 points, and the mean QMGS after treatment was 17.93 ± 5.61 points, and the score decreased by 5.47 ± 4.16 points after treatment, and the difference was statistically significant (Figure 2A, *p* < 0.001). Taking a 3-point or more drop in the post-treatment score as the criterion for effective treatment, we found that 75.6% (93/123) of patients had an improvement in clinical symptoms. And 24.4% (30/123) of patients responded poorly to treatment (QMGS improvement < 3 points), including 4 (3.3%) patients with worse QMGS after treatment and 3 (2.4%) patients with myasthenic crisis after treatment. Ten (8.1%) patients received additional IVIG therapy. There were no patient deaths throughout the course of treatment.

In addition, we further analyzed changes in patients' QMGS from baseline to 14, 30, and 60 days after completing treatment. Due to lack of complete QMGS follow-up data, 59 patients were excluded from this analysis and 64 patients were included. The results showed that the QMGS of patients demonstrated a significant improvement trend after LPE (Figure 2B, *p* < 0.001). The mean QMGS was 24.03 ± 4.34 points before treatment, 15.89 ± 5.58 points 14 days after completing treatment, 14.42 ± 5.99 points 30 days after completing treatment, and 11.56 ± 6.14 points 60 days after completing treatment.

### Sensitivity of different involved muscle groups to treatment

We further explored whether different muscle groups involved in MG differ in their responsiveness to treatment. The mean QMGS of ocular muscles was 5.22 ± 1.91 points before treatment, and was 4.01 ± 2.11 points after treatment, a decrease of 1.21 ± 1.58 points, and the difference was statistically significant (Figure 2C, *p* < 0.001). The mean score of limb muscles was 13.05 ± 3.86 points before treatment, and was 10.83 ± 4.07 points after treatment, with a difference of 2.22 ± 2.56 points, which was significant (Figure 2D, *p* < 0.001). For the bulbar muscles, the mean score was 2.59 ± 1.96 points before treatment and 1.29 ± 1.60 points after treatment, which decreased by 1.30±1.37 points, and the difference was statistically significant (Figure 2E, *p* < 0.001). For respiratory muscles, the mean QMGS decreased from 2.54 ± 0.83 points before treatment to 1.73 ± 1.13 points after treatment, a statistically significant decrease of 0.81 ± 0.98 points (Figure 2F, *p* < 0.001).

### Effects of LPE on serum autoantibody and inflammatory factors

**Figure 3:**
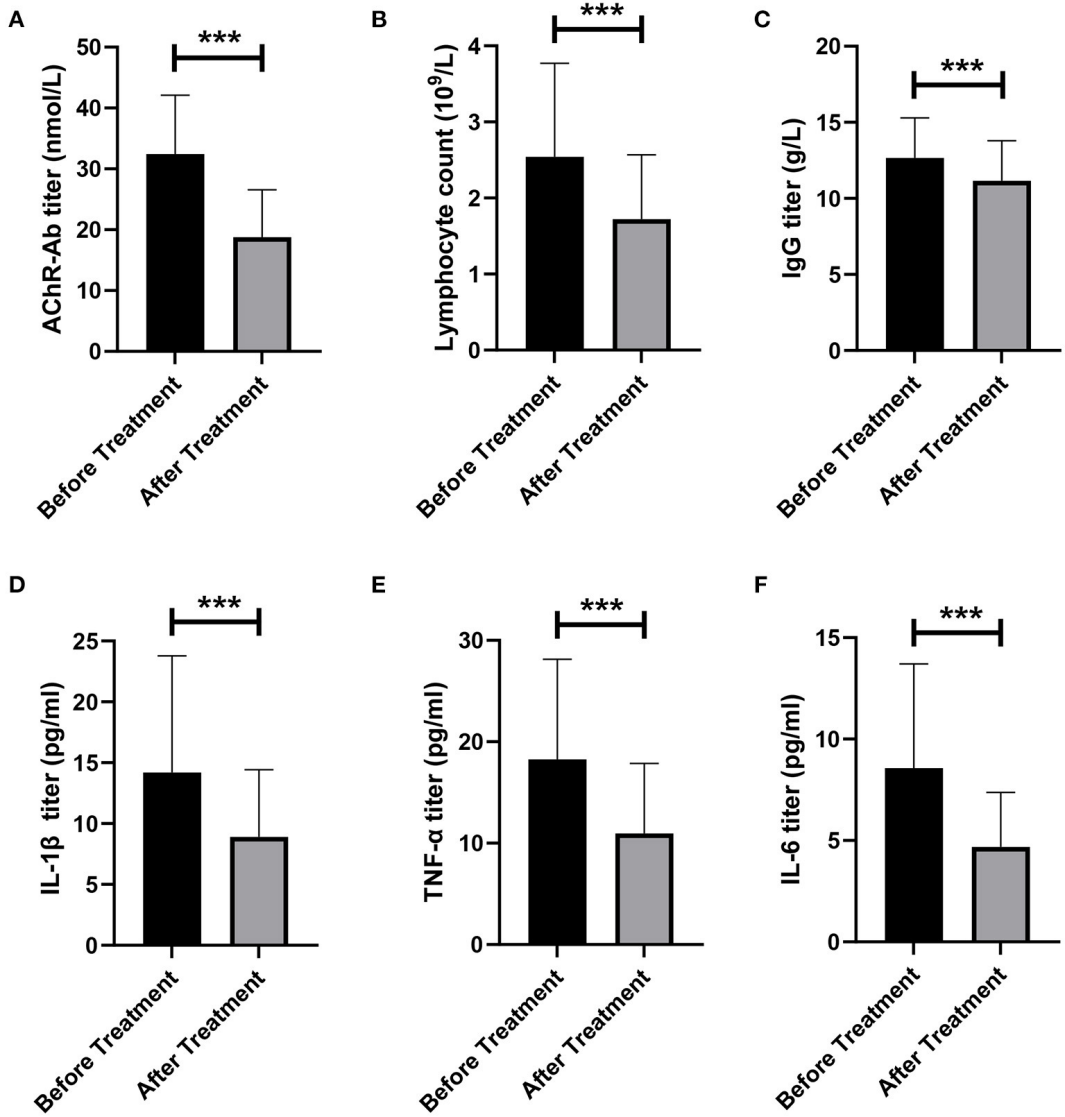
Changes of immune indexes before and after treatment. **(A)** Changes of AChR-Ab titer before and after treatment. **(B)** Changes in the number of lymphocytes before and after treatment. **(C)** Changes in IgG titer before and after treatment. **(D–F)** Changes of proinflammatory factors IL-1β **(D)**, TNF-α **(E)**, and IL-6 **(F)** before and after treatment. ***p < 0.001.

We evaluated the effect of LPE on immune-related indicators in patients. We found that AChR-Ab level in patients decreased significantly after treatment (32.45 ± 9.66 vs. 18.78 ± 7.78 noml/L, *p* < 0.001, Figure 3A). The lymphocyte counts and IgG level after treatment were also significantly lower than those before treatment (lymphocytes, 2.54 ± 1.23 vs. 1.72 ± 0.85 × 10^∧^9/L, *p* < 0.001; IgG, 12.66 ± 2.63 vs. 11.15 ± 2.64 g/L, *p* < 0.001, Figures 3B,C). In addition, we assessed the changes of proinflammatory cytokines before and after treatment. The results showed that LPE significantly reduced the levels of IL-1β, TNF-α, and IL-6 (IL-1β, 14.19 ± 9.59 vs. 8.90 ± 5.53 pg/ml, *p* < 0.001; TNF-α, 18.26 ± 9.87 vs. 10.96 ± 6.86 pg/ml, *p* < 0.001; IL-6, 8.57 ± 5.14 vs. 4.68 ± 2.69 pg/ml, *p* < 0.001, Figures 3D–F).

### Clinical factors associated with treatment outcome

**Figure 4:**
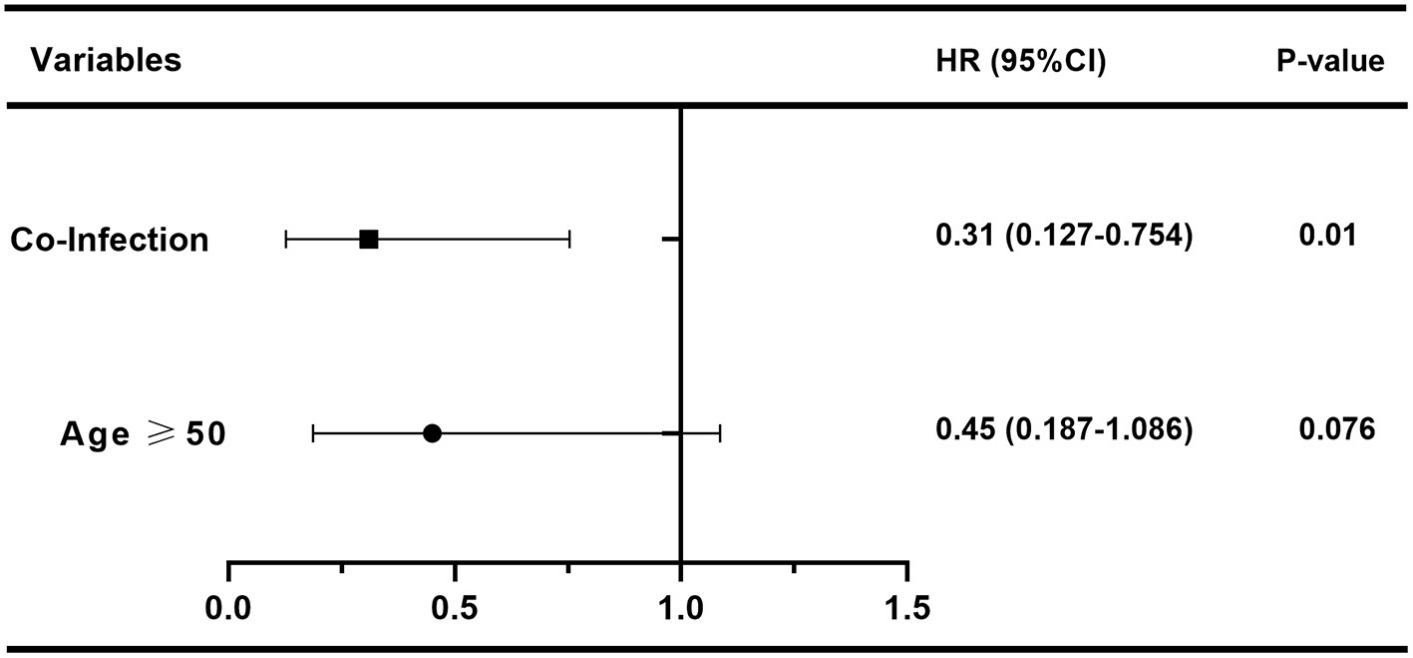
Forest plot of multivariate logistic regression analysis results.

In order to assess the effect of baseline clinical characteristics on treatment outcome, we performed logistic regression analysis using the effective treatment (QMGS reduction ≥3 points) as the outcome measure. In this analysis, we stratified for age: ≥50 and <50 years. In univariate logistic regression analysis, we found that gender, disease duration, Ab status, thymus condition, thymectomy, immunotherapy before treatment, history of myasthenic crisis, coexistence with other autoimmune disorders, MGFA classification, and baseline QMGS were not associated with treatment response (Table 2, *p* > 0.05). And age ≥50 years (HR 0.383, 95% CI 0.163–0.896, *p* = 0.027) and infection (HR 0.274, 95% CI 0.115–0.654, *p* = 0.004) were unfavorable factors affecting efficacy (Table 2). We further included age and infection in multivariate logistic regression and found that infection was an independent risk factor for adverse treatment outcomes (HR 0.31, 95% CI 0.127–0.754, *p* = 0.01, Figure 4).

**Table 2:** Univariate logistic regression analysis of factors affecting LPE efficacy.

	**HR**	**95% CI**	***P*** **-value**
Age ≥ 50 years	0.383	0.163–0.896	0.027^*^
Gender (male)	0.629	0.273–1.447	0.275
Course of disease	1.003	0.996–1.010	0.401
Thymoma	1.027	0.368–2.868	0.959
Thymic hyperplasia	0.967	0.097–9.657	0.977
Thymectomy	0.753	0.292–1.945	0.558
Coexistence with other autoimmune diseases	2.625	0.724–9.517	0.142
Co-infection	0.274	0.115–0.654	0.004^**^
Immunotherapy before treatment	1.214	0.533–2.768	0.644
History of myasthenic crisis	1.207	0.313–4.651	0.784
MGFA classification (IVb)	1.203	0.423–3.418	0.729
Baseline QMG score	0.980	0.890–1.080	0.690
AChR-Ab	0.532	0.111–2.551	0.430
MuSK-Ab	0.967	0.097–9.657	0.977
Titin-Ab	0.660	0.253–1.722	0.396
RyR-Ab	5.139	0.647–40.846	0.122

HR, Hazard ratio; 95% CI, 95% confidence interval;

* p < 0.05,

** p < 0.01.

### Safety of LPE

In this study cohort, LPE demonstrated a good safety profile with mild adverse effects and no serious adverse events. Among 123 patients, 13% (*n* = 16) of patients experienced adverse reactions. Citrate reaction caused by hypocalcemia and allergic symptoms were common, such as skin pruritus, rash, dyspnea, chest tightness, facial flushing, tetany, and numbness of limbs, which can be effectively relieved after symptomatic treatment. No serious adverse events such as hemorrhage, circulatory overload, shock, hypothermia, and hemolysis were observed during or after LPE treatment. No patients developed vascular pathway-related infections, and no exacerbation of pre-existing infections due to LPE was observed. There were no adverse reactions leading to patient death. A summary of LPE-related adverse reactions was presented in Table 3.

**Table 3:** Adverse reactions associated with LPE.

**Adverse events**	***n*** **(%)**
Skin pruritus	8 (6.5)
Rash	8 (6.5)
Tetany	2 (1.6)
Dyspnea	1 (0.8)
Chest tightness	1 (0.8)
Facial flushing	1 (0.8)
Numbness of limbs	1 (0.8)

## Discussion

In this study, we explored the efficacy and safety of LPE in severe MG through a retrospective analysis of clinical data. We enrolled 123 patients with severe MG who received LPE and found a significant improvement in QMGS after treatment. In terms of the proportion of patients who benefited, about three-quarters had a good response to the treatment. In addition, we found that all muscle groups involved in MG were sensitive to LPE treatment, with significant improvement in scores. Studies have shown that the duration of PE efficacy is about 1–2 months (22, 23). This study also found that the patients' QMGS gradually improved during the 2-month follow-up. However, the results of QMGS changes during follow-up should be interpreted with caution, because the patients also received long-term immunomodulatory therapy. Since corticosteroids and immunosuppressants usually take weeks and months to manifest beneficial effects (9, 23, 24), we believe that the impact of long-term immunotherapy on the short-term efficacy of LPE is very limited, but the extent to which it amplifies our judgment of the LPE efficacy over a longer observation period remains to be further determined. In this study cohort, LPE was well tolerated with mild adverse reactions, mainly anaphylactic reactions and hypocalcemia-induced citrate reaction, which is consistent with previous studies (11, 18). And further studies are needed to clarify whether prophylactic administration of calcium gluconate and antiallergic drugs is necessary before treatment. Overall, LPE demonstrated good efficacy and safety for severe MG in this study cohort.

Furthermore, we found that the levels of AChR-Ab and pro-inflammatory cytokines IL-1β, IL-6, and TNF-α decreased significantly in patients after treatment. As the main pathogenic antibody for MG, a decrease in AChR-Ab titer was associated with clinical improvement in patients (25). IL-1β induces naive CD4^+^ T cells to differentiate into T helper 17 (Th17) cells (26), which express the transcription factor retinoic acid-related orphan receptor γt (RORγt), release various cytokines such as IL-17 and IL-22, and activate immune cells. Th17 cells and related cytokines are associated with chronic inflammation of neuromuscular junction and play an important role in the immunological pathogenesis of MG (27). IL-6 is an important factor regulating autoimmune responses in MG. IL-6 has multiple biological activities related to inflammation, including potent B cell stimulation and differentiation, T cell activation, antibody production (27), and plays a vital role in immune imbalance between Th17 cells and regulatory T cells (Tregs) (28). TNF-α is considered to be an important pro-inflammatory cytokine in T cell-mediated autoimmune diseases (29), and its biological effects in MG include induction of IL-6, activation of B cells, and priming of AChR-specific T cells (30).

Compared with traditional PE, LPE may have its unique advantages in the mechanism of action. The removal of autoantibodies, inflammatory factors and other pathological substances in plasma by traditional PE may relieve the feedback inhibition of immunocompetent cells, resulting in a rebound in the level of immune pathogenic factors (11). LPE can not only eliminate soluble pathogenic factors in plasma, but also remove sensitized immunocompetent cells, preventing the continuous production of pathological factors, so that the pathological immune responses can be controlled more effectively and lastingly (11, 15, 16, 18). In this study, we also found that the level of lymphocytes decreased in patients after treatment, but the effect of LPE on immune cell subsets is still ambiguous, and further studies are needed to clarify.

At present, there is still a lack of studies comparing the efficacy of LPE and traditional PE in MG. We reviewed previous high-quality studies evaluating the efficacy of PE. In a randomized, evaluator-masked trial involving 43 patients with moderate-to-severe MG who received PE, with the change in QMGS at day 14 after treatment as the primary outcome measure, the QMGS of patients decreased by 4.7 ± 4.9 points, and 57% of patients responded to PE therapy (31). In another randomized clinical trial involving 41 patients with MG exacerbation, the response rate for PE was 66% (32). In this study, the improvement rate of patients was 75.6%, and the magnitude of QMGS decline was 5.47 ± 4.16 points. Given the retrospective nature of this study and the lack of controls, it is currently difficult to assess whether there is a difference in the efficacy of LPE and PE in severe MG. However, it's worth noting that patients in this study cohort received an average of only 1.79 replacements. Compared with traditional PE (standard protocol: 5 replacement sessions over 2 weeks), the number of replacement sessions is significantly reduced, which not only shortens the length of hospital stay, cuts down plasma consumption and treatment burden, but also reduces the risk of serious adverse reactions during multiple replacements.

In this cohort, 24.4% of patients had co-infections prior to treatment, with respiratory infections being the most common type of infection, consistent with previous findings (33). Patients with MG are at increased risk of infection, which may be a direct effect of immunosuppression during immunomodulatory therapy, and the patient's own immune disorders may also play a role (34). Infection is not only an important driver of disease deterioration in patients with MG (35, 36), but also an adverse factor affecting the prognosis of MG treatment (37). In this study, we also found that infection was detrimental to patients' response to LPE. Therefore, the avoidance and rapid and effective control of infection are necessary for patients with MG. In addition, we found that age was a possible factor affecting treatment outcome, and older patients may have a poorer response to treatment. The effect of age on the treatment of MG is currently unclear. Studies have shown that elderly patients have lower treatment remission rates than younger patients (38), and that older age is associated with adverse outcomes in MG (39). However, some studies have also found that younger patients are more likely to exhibit drug-refractory MG than older patients (40, 41).

In general, our study suggests that LPE may be an effective and safe treatment option for severe MG. Of course, our research also has some shortcomings. First of all, this study was retrospective and selection bias cannot be ruled out. Secondly, this study was single-center, and the results may be influenced by institution-specific practices. Furthermore, the relatively limited sample size of this study and the loss of some patients during follow-up may lead to biased results. Finally, the lack of PE control group makes it impossible to evaluate whether LPE is superior to PE in efficacy. The results of this study need further randomized controlled studies to verify.

## Data availability statement

The original contributions presented in the study are included in the article/Supplementary material, further inquiries can be directed to the corresponding author.

## Ethics statement

The studies involving human participants were reviewed and approved by Ethics Committee of Xiangya Hospital. The patients/participants provided their written informed consent to participate in this study.

## Author contributions

HY conceived and designed the research. XD, YL, HC, and QZ collected the clinical data. WD and HZ analyzed the data. WD, SO, and WY drafted the manuscript. BL performed the lymphoplasmapheresis. All authors contributed to the article and approved the submitted version.

## Funding

This research was supported by the National Natural Science Foundation of China (81771364 and 82171399), Science and Technology Innovation Guidance Project of Hunan Province (2020SK53009), Changsha Municipal Natural Science Foundation (kq2007037), and Natural Science Foundation of Hunan Province (2022JJ40724).

## Conflict of interest

The authors declare that the research was conducted in the absence of any commercial or financial relationships that could be construed as a potential conflict of interest.

## Publisher's note

All claims expressed in this article are solely those of the authors and do not necessarily represent those of their affiliated organizations, or those of the publisher, the editors and the reviewers. Any product that may be evaluated in this article, or claim that may be made by its manufacturer, is not guaranteed or endorsed by the publisher.

## References

[1] Carr AS Cardwell CR McCarron PO McConville J. A systematic review of population based epidemiological studies in myasthenia gravis. BMC Neurol. (2010) 10:46. 10.1186/1471-2377-10-46 20565885 PMC2905354

[2] Albazli K Kaminski HJ Howard JF. Complement inhibitor therapy for myasthenia gravis. Front Immunol. (2020) 11:917. 10.3389/fimmu.2020.00917 32582144 PMC7283905

[3] Lazaridis K Tzartos SJ. Myasthenia gravis: autoantibody specificities and their role in Mg management. Front Neurol. (2020) 11:596981. 10.3389/fneur.2020.596981 33329350 PMC7734299

[4] Xiao H Wu K Liang X Li R Lai KP. Clinical efficacy and safety of eculizumab for treating myasthenia gravis. Front Immunol. (2021) 12:715036. 10.3389/fimmu.2021.715036 34456922 PMC8384962

[5] Keller CW Pawlitzki M Wiendl H Lünemann JD. Fc-Receptor targeted therapies for the treatment of. Int J Mol Sci. (2021) 22:5755. 10.3390/ijms22115755 34071155 PMC8198115

[6] Melzer N Ruck T Fuhr P Gold R Hohlfeld R Marx A. Clinical features, pathogenesis, and treatment of myasthenia gravis: a supplement to the guidelines of the German neurological society. J Neurol. (2016) 263:1473–94. 10.1007/s00415-016-8045-z 26886206 PMC4971048

[7] Sanders DB Wolfe GI Benatar M Evoli A Gilhus NE Illa I. International consensus guidance for management of myasthenia gravis: executive summary. Neurology. (2016) 87:419–25. 10.1212/WNL.0000000000002790 27358333 PMC4977114

[8] Narayanaswami P Sanders DB Wolfe G Benatar M Cea G Evoli A. International consensus guidance for management of myasthenia gravis: 2020 update. Neurology. (2021) 96:114–22. 10.1212/WNL.0000000000011124 33144515 PMC7884987

[9] Alhaidar MK Abumurad S Soliven B Rezania K. Current treatment of myasthenia gravis. J Clin Med. (2022) 11:1597. 10.3390/jcm11061597 35329925 PMC8950430

[10] Mantegazza R Antozzi C. From traditional to targeted immunotherapy in myasthenia gravis: prospects for research. Front Neurol. (2020) 11:981. 10.3389/fneur.2020.00981 32982957 PMC7492201

[11] Zhang Z Yuan X Jiang Y Li N Li B. Effectiveness of lymphoplasmapheresis compared with therapeutic plasma exchange for thrombotic thrombocytopenic purpura: a retrospective evaluation. Hematology. (2022) 27:167–72. 10.1080/16078454.2021.2015842 35068383

[12] Wallace D Goldfinger D Lowe C Nichols S Weiner J Brachman M. A double-blind, controlled study of lymphoplasmapheresis versus sham apheresis in rheumatoid arthritis. N Engl J Med. (1982) 306:1406–10. 10.1056/NEJM198206103062307 7043264

[13] Wallace DJ Medici MA Nichols S Klinenberg JR Bick M Gatti R. Plasmapheresis versus lymphoplasmapheresis in rheumatoid arthritis: immunologic comparisons and literature review. J Clin Apher. (1984) 2:184–9. 10.1002/jca.2920020207 6536669

[14] Kleinman S Nichols M Strauss F Goldfinger D. Use of lymphoplasmapheresis or plasmapheresis in the management of acute renal allograft rejection. J Clin Apher. (1982) 1:14–7. 10.1002/jca.2920010105 6765451

[15] Zhang M Zhang Y Zhu W Kuang Y. Successful use of lymphoplasma exchange in a patient with acute generalized pustular psoriasis of von zumbusch. Dermatol Ther. (2020) 33:e14092. 10.1111/dth.14092 32720411

[16] Wang B Li J Xie HF Chen M Li B Shi W. Striking case of febrile ulceronecrotic mucha-habermann disease responding to lymphoplasmapheresis and methotrexate. J Dermatol. (2020) 47:e430–1. 10.1111/1346-8138.15598 32940361

[17] Luo MC Wang WF Yin WF Li Y Li BJ Duan WW. Clinical efficacy and mechanism of lymphoplasma exchange in the treatment of guillain-barre syndrome. Cell Mol Biol. (2017) 63:106–15. 10.14715/cmb/2017.63.10.17 29096750

[18] Zhang L Zhuang Y Liu X Xu Q Zhou L Zou L. The efficacy of therapeutic apheresis in patients with refractory neuromyelitis optica spectrum disorders: a single-center retrospective study. Ann Palliat Med. (2021) 10:3105–14. 10.21037/apm-21-177 33752428

[19] Gilhus NE. Myasthenia gravis. N Engl J Med. (2016) 375:2570–81. 10.1056/NEJMra1602678 28029925

[20] Jaretzki A Barohn RJ Ernstoff RM Kaminski HJ Keesey JC Penn AS. Myasthenia gravis: recommendations for clinical research standards. Task force of the medical scientific advisory board of the myasthenia gravis foundation of America. Neurology. (2000) 55:16–23. 10.1212/WNL.55.1.16 10891897

[21] Barohn RJ McIntire D Herbelin L Wolfe GI Nations S Bryan WW. Reliability testing of the quantitative myasthenia gravis score. Ann N Y Acad Sci. (1998) 841:769–72. 10.1111/j.1749-6632.1998.tb11015.x 9668327

[22] Sieb JP. Myasthenia gravis: an update for the clinician. Clin Exp Immunol. (2014) 175:408–18. 10.1111/cei.12217 24117026 PMC3927901

[23] Gilhus NE Verschuuren JJ. Myasthenia gravis: subgroup classification and therapeutic strategies. Lancet Neurol. (2015) 14:1023–36. 10.1016/S1474-4422(15)00145-3 26376969

[24] Morren J Li Y. Maintenance immunosuppression in myasthenia gravis, an update. J Neurol Sci. (2020) 410:116648. 10.1016/j.jns.2019.116648 31901719

[25] Oosterhuis HJ Limburg PC Hummel-Tappel E The TH. Anti-Acetylcholine receptor antibodies in myasthenia gravis. Part 2. Clinical and serological follow-up of individual patients. J Neurol Sci. (1983) 58:371–85. 10.1016/0022-510X(83)90096-5 6842265

[26] Villegas JA Van Wassenhove J Le Panse R Berrih-Aknin S Dragin N. An imbalance between regulatory T cells and T helper 17 cells in acetylcholine receptor-positive myasthenia gravis patients. Ann N Y Acad Sci. (2018) 1413:154–62. 10.1111/nyas.13591 29405352

[27] Uzawa A Kuwabara S Suzuki S Imai T Murai H Ozawa Y. Roles of cytokines and T cells in the pathogenesis of myasthenia gravis. Clin Exp Immunol. (2021) 203:366–74. 10.1111/cei.13546 33184844 PMC7874834

[28] Chen P Tang X. Gut microbiota as regulators of Th17/Treg balance in patients with myasthenia gravis. Front Immunol. (2021) 12:803101. 10.3389/fimmu.2021.803101 35003133 PMC8732367

[29] Duan R-S Wang H-B Yang J-S Scallon B Link H Xiao B-G. Anti-Tnf-Alpha antibodies suppress the development of experimental autoimmune myasthenia gravis. J Autoimmun. (2002) 19:169–74. 10.1006/jaut.2002.0618 12473237

[30] Tüzün E Huda R Christadoss P. Complement and cytokine based therapeutic strategies in myasthenia gravis. J Autoimmun. (2011) 37:136–43. 10.1016/j.jaut.2011.05.006 21636248

[31] Barth D Nabavi Nouri M Ng E Nwe P Bril V. Comparison of Ivig and plex in patients with myasthenia gravis. Neurology. (2011) 76:2017–23. 10.1212/WNL.0b013e31821e5505 21562253 PMC3109880

[32] Gajdos P Chevret S Clair B Tranchant C Chastang C. Clinical trial of plasma exchange and high-dose intravenous immunoglobulin in myasthenia gravis. Myasthenia gravis clinical study group. Ann Neurol. (1997) 41:789–96. 10.1002/ana.410410615 9189040

[33] Kassardjian CD Widdifield J Paterson JM Kopp A Nagamuthu C Barnett C. Serious infections in patients with myasthenia gravis: population-based cohort study. Eur J Neurol. (2020) 27:702–8. 10.1111/ene.14153 31997519

[34] Prior DE Nurre E Roller SL Kline D Panara R Stino AM. Infections and the relationship to treatment in neuromuscular autoimmunity. Muscle Nerve. (2018) 57:927–31. 10.1002/mus.26032 29211921 PMC5951723

[35] Gummi RR Kukulka NA Deroche CB Govindarajan R. Factors associated with acute exacerbations of myasthenia gravis. Muscle Nerve. (2019) 60:693–9. 10.1002/mus.26689 31469909

[36] Lizarraga AA Lizarraga KJ Benatar M. Getting rid of weakness in the ICU: an updated approach to the acute management of myasthenia gravis and Guillain-Barré syndrome. Semin Neurol. (2016) 36:615–24. 10.1055/s-0036-1592106 27907966

[37] Liu Z Yao S Zhou Q Deng Z Zou J Feng H. Predictors of extubation outcomes following myasthenic crisis. J Int Med Res. (2016) 44:1524–33. 10.1177/0300060516669893 27856933 PMC5536745

[38] Donaldson DH Ansher M Horan S Rutherford RB Ringel SP. The relationship of age to outcome in myasthenia gravis. Neurology. (1990) 40:786–90. 10.1212/WNL.40.5.786 2330105

[39] Basta I Pekmezović T Peric S Nikolić A Rakočević-Stojanović V Stević Z. Survival and mortality of adult-onset myasthenia gravis in the population of belgrade, Serbia. Muscle Nerve. (2018) 58:708–12. 10.1002/mus.26132 29572981

[40] Cortés-Vicente E Álvarez-Velasco R Segovia S Paradas C Casasnovas C Guerrero-Sola A. Clinical and therapeutic features of myasthenia gravis in adults based on age at onset. Neurology. (2020) 94:e1171–80. 10.1212/WNL.0000000000008903 32071167 PMC7220233

[41] Cortés-Vicente E Álvarez-Velasco R Pla-Junca F Rojas-Garcia R Paradas C Sevilla T. Drug-Refractory myasthenia gravis: clinical characteristics, treatments, and outcome. Ann Clin Transl Neurol. (2022) 9:122–31. 10.1002/acn3.51492 35080153 PMC8862423

