# Supplementary material for: Application of lymphoplasmapheresis in the treatment of severe myasthenia gravis
Source: Front Neurol. 2022 Oct 11;13:1018509. doi: 10.3389/fneur.2022.1018509 (PMC9595276; doi:10.3389/fneur.2022.1018509)
Supplement: Supplementary file 1 [file Table_1.DOCX]

Supplementary Material

# Supplementary Table

**Table S1.** Myasthenia Gravis Foundation of America (MGFA) clinical classification.

| **Class I** | Ocular myasthenia gravis |
| --- | --- |
| **Class II** | Mild weakness affecting other than ocular muscles |
| Class IIa | Predominantly affecting limb and axial muscles |
| Class IIb | Predominantly affecting oropharyngeal and respiratory muscles |
| **Class III** | Moderate weakness affecting other than ocular muscles |
| Class IIIa | Predominantly affecting limb and axial muscles |
| Class IIIb | Predominantly affecting oropharyngeal and respiratory muscles |
| **Class IV** | Severe weakness affecting other than ocular muscle |
| Class IVa | Predominantly affecting limb and axial muscles |
| Class IVb | Predominantly affecting oropharyngeal and respiratory muscles |
| **Class V** | Defined by intubation, with or without mechanical ventilation, except when employed during routine postoperative management. |
